# Internet addiction and depressive symptoms in adolescents: joint trajectories and predictors

**DOI:** 10.3389/fpubh.2024.1374762

**Published:** 2024-06-04

**Authors:** Junjie Zhang, Enna Wang, Long Zhang, Xinli Chi

**Affiliations:** ^1^Collaborative Innovation Center of Assessment for Basic Education Quality, Beijing Normal University, Beijing, China; ^2^School of Psychology, Northwest Normal University, Lanzhou, China; ^3^Faculty of Humanities and Social Sciences, City University of Macau, Macau, China; ^4^Mental Health Education Center, Yunnan College of Business Management, Kunming, Yunnan, China; ^5^School of Psychology, Shenzhen University, Shenzhen, China; ^6^The Shenzhen Humanities and Social Sciences Key Research Bases of the Center for Mental Health, Shenzhen University, Shenzhen, China

**Keywords:** joint trajectories, Internet addiction, depressive symptoms, adolescents, longitudinal study

## Abstract

**Objective:**

Internet addiction and depressive symptoms are common mental health problems in adolescents. Due to the comorbidity of Internet addiction and depressive symptoms, their mutual relationship influences their developmental trajectories over time. Thus, this study aimed to identify the joint trajectories of Internet addiction and depressive symptoms, and examined the individual, family, and school antecedents of these trajectories among Chinese adolescents.

**Methods:**

Using a battery of self-report scales, three waves of data collection were conducted in a Chinese adolescent sample (*N* = 1,301). The co-developmental trajectories of Internet addiction and depressive symptoms were extracted by adopting parallel-process latent class growth modeling (PPLCGM). Multinomial logistic regression was performed to assess predictive factors.

**Results:**

Four unique joint trajectory classes were detected: the Health Group (*n* = 912, 70.1%), Comorbidity-Worsening Group (*n* = 85, 6.5%), Asymptomatic-Comorbid Risk Group (*n* = 148, 11.4%), and Prominent Depressive Symptoms-Remission Group (*n* = 156, 12.0%). Individual, family, and school factors (e.g., gender, positive youth development, family function, academic performance) significantly predicted the membership in these distinct co-developmental trajectories.

**Conclusion:**

Our findings illustrate that the joint development of Internet addiction and depressive symptoms among adolescents presents a heterogeneous distribution, which could better inform prevention and intervention strategies since each co-developmental trajectory may represent unique experience for adolescents who need targeted treatment. Various individual, family, and school factors are important predictors that play different roles in distinguishing the joint trajectories of Internet addiction and depressive symptoms during this critical developmental transition period.

## Introduction

Internet addiction is defined as “excessive or uncontrolled urges in terms of Internet use” (1). Currently, adolescent Internet addiction has become a prime concern across countries and regions. The prevalence of Internet addiction among adolescents has been shown to vary greatly from 11.8% (European countries) to 20.6% (Canada) (2). In China, Internet addiction rates have been reported to reach 15.3% among adolescents (3). Internet addiction is associated with a range of detrimental physical and mental health issues, including sleep disturbances (4), emotional distress (5, 6), and poor interpersonal relationships (7). Characterized by “feelings of sadness and worthlessness, with diminished interest in things which were once enjoyed, and even having suicide ideation” (8), depressive symptoms are one of the most common correlates of Internet addiction (9, 10), making it necessary to survey this particularly relevant construct in conjunction with being addictive into the Internet. Epidemiological research has also frequently revealed that there is a high incidence of comorbidity between these disturbances (11, 12), and this comorbidity often results in a more chronic course and more severe psychiatric symptoms, a higher recurrence rate, and heavier burdens than single diseases (13, 14).

Theoretically, the relationship between Internet addiction and depressive symptoms should be reciprocal: based on the compensatory Internet use theory (15), adolescents who become depressed are more likely to engage in the Internet world, come in contact with virtual friends to alleviate negative emotions and compensate for psychological needs; meanwhile, according to the social displacement hypothesis, overindulgence into the Internet may usurp face-to-face communication and result in social isolation, thus heightening the chances of suffering from depression (16). Moreover, recent empirical studies that explored the temporal directional relationship between Internet addiction and depressive symptoms also supported the idea that Internet addiction and depressive symptoms reciprocally influence each other (17, 18). Although Internet addiction and depressive symptoms are theoretically and empirically entwined, the developmental overlap between Internet addiction developmental trajectories and depression developmental trajectories still remains unclear. Consequently, this study anticipated to probe their joint developmental courses among Chinese adolescents during their junior high school years. In addition, consecrating that the explanation for the high prevalence of comorbidities between Internet addiction and depressive symptoms among adolescents might be the basis of the etiopathogenesis and influencing factors shared between them, we also examined whether a series of individual, school and family factors were differentially associated with the longitudinal joint trajectory classes.

### Distinct trajectories of Internet addiction and depressive symptoms among adolescents

A handful of studies have observed the evolution of Internet addiction. Specifically, using growth curve analyses, several researchers have shown that Internet addiction tends to increase as children enter puberty, but is initially moderate and subsequently decreases when adolescents reach the age range from 16 to 19 (19). However, these studies using latent curve modeling mainly displayed general developmental trends but ignored the heterogeneously distributed trajectories of adolescent Internet addiction, which might yield results of limited interpretability. This issue could be solved by adopting growth mixture models or latent class growth models for longitudinal data (20), which allows us to examine whether populations could be qualitatively differentiated into latent subgroups with distinct developmental trajectories.

Limited studies have examined the heterogeneous growth trajectories of Internet addiction over time (21–24). These studies identified four to six trajectory subgroups of Internet addiction. For example, Zhou et al. (24) identified four developmental trajectory subgroups of Internet addiction among youth experiencing the Wenchuan earthquake: a slowly rising group, a steadily rising group, a sharply falling group, and a sharply rising group. In another study, a sample of 3,079 Singaporean youth aged 7 to 19 years was longitudinally surveyed four times at one-year intervals, revealing six latent trajectory types across this period based on changes in Internet addiction related to pathological status (21).

Many studies have examined heterogeneous longitudinal patterns of depressive symptoms from childhood to adolescence (25–30). Typically, scholars have identified three to four distinct developmental trajectories of depressive symptoms over time. Ellis et al. (26) had reviewed 18 studies that examined the developmental heterogeneity of adolescent depression and found that previous studies reported a low-level stable trajectory of adolescent depression, and most of them also found both an increasing and a decreasing trajectory. Recent studies have confirmed these conclusions. For example, Ho et al. (31) also identified three trajectories—a “high but decreasing trajectory,” a “moderate and stable trajectory,” and a “low but increasing trajectory”—in adolescents through a three-year follow-up study.

These studies mentioned above highlight the importance of the longitudinal development of Internet addiction and depressive symptoms in adolescents. Nevertheless, there are still limitations that have not yet been addressed: most studies exclusively concern the heterogeneous development trajectories of either Internet addiction or depressive symptoms but do not pay close attention to the longitudinal overlap between them. In fact, researchers have reached the consensus that Internet addiction is often comorbid with depressive symptoms (10). Thus, the trajectories of adolescent Internet addiction might influence, and be influenced by depressive symptoms. Thus, it is expected that the two mental health issues will exhibit a similar developmental course. Examining the joint growth trajectories of Internet addiction and depressive symptoms is important for obtaining a better understanding of how these developmental courses evolve from early adolescence to middle adolescence. These issues are also crucial for establishing effective prevention or intervention strategies that consider the developmental relationships between Internet addiction and depressive symptoms. Consequently, the first aim of the present study was to investigate the joint development of Internet addiction and depressive symptoms among Chinese adolescents.

### Predictive factors associated with Internet addiction and depressive symptoms

Substantial studies have reported on the predictive factors associated with Internet addiction and depressive symptoms (22, 31–33). In this study, based on the results of our previous studies on the prevalence and predictors of Internet addiction and depressive symptoms (3, 34), we selected a set of robust predictors for the current study, and those influential factors were classified as individual, family, and school variables.

Regarding individual factors, gender and positive youth development were chosen. The findings on gender differences have been mixed. Some studies have revealed that boys are usually more likely to get indulged into Internet than are their female peers (3, 35). However, because girls are more sensitive to the environment, they are at greater risk of experiencing depressive symptoms (36). There were also studies observed non-significant gender differences with respect to both Internet addiction and depressive symptoms (34, 37, 38). Different from gender, positive youth development has been discovered to be a robust predictor of both Internet addiction (39, 40) and depressive symptoms (41, 42). Researchers have consistently suggested that adolescents with low levels of positive youth development project the image of having fewer resources to effectively defend themselves, facilitating the development of problem behaviors and emotional disorders (43–47).

Concerning family factors, family structure, whether adolescents came from an only-child family or not, and family function were selected. To a greater extent, family non-intactness, encompassing divorce, separation, or remarriage, has also been a contributor to adolescent Internet addiction (48) and depressive symptoms (34). For instance, Ni et al. (49) suggested that having a single-parent family was significantly associated with Internet addiction. This is also suitable for adolescents’ depressive symptoms (50). Due to the implementation of the one-child policy in China during the preceding three decades, a significant number of families have been limited to having only one child. Extensive research has been conducted to examine potential disparities in psychological and behavioral development between children raised in single-child households and those from families with multiple children (51–53). Research has demonstrated that there are significant differences in Internet addiction (54) and depressive symptoms (34) between only children and non-only children in the Chinese adolescent population. Furthermore, given the importance of family as a source of emotional support and emotional warmth for adolescents, it should be no surprise that healthy family function has been verified to be a prominent protective factor against adolescents’ Internet addiction (55) and depressive symptoms (56). Adolescents who report family dysfunction issues, such as lack of communication, frequent conflict and indifferent atmospheres, have been found to be at high risk of being addictive toward the Internet (57) and becoming depressed (58). Thus, the adolescents’ perceptions of family function were also assessed.

As for school factors, academic performance was chosen. According to the academic incompetence hypothesis (59), inadequate academic performance or failure in academics could potentially diminish intrinsic resources among adolescents, thereby reducing their capacity to effectively manage addiction and depression. Empirical research likewise suggests that poor academic performance or academic failure may lead to the development of Internet addiction (60) and depressive symptoms (61). Specifically, the occurrence of substandard academic performance has the potential to trigger a range of adverse cognitive processes, such as diminished self-esteem (62), and negative interpersonal encounters, such as peer rejection (63), which have also been linked to an increased likelihood of Internet addiction and depressive symptoms.

These aforementioned findings suggest that both the Internet addiction and depressive symptoms of adolescents are influenced by various factors present within the ecosystem in which adolescents live. Nevertheless, it remains unclear whether these predictors might play the same role in the joint trajectory of Internet addiction and depressive symptoms. Failure to take the developmental trajectory and heterogeneity into account may generate limited generalizability and interpretability since different etiologic factors might operate to produce different levels of severity or different developmental trajectories of problems. Therefore, in the present study, we also examined a range of variables that were selected to include different domains termed individual variables (gender, positive youth development), family variables (single-child family, family function), and school variables (academic achievement). We aimed to provide an overall picture of what factors could predict membership in the conjoint trajectory groups of Internet addiction and depressive symptoms, which was the second aim of our study.

### The present study

Overall, the present study aimed to explore (1) the joint developmental trajectories of Internet addiction and depressive symptoms among Chinese adolescents and (2) the individual, family, and school factors related to membership in these joint trajectories of Internet addiction and depressive symptoms among adolescents. Since no study to date has examined the joint trajectory classes of Internet addiction and depressive symptoms and the predictors of these classes, in this study, both of the goals were exploratory in nature, and no specific hypotheses were put forward. The present study is expected to help practitioners offer tailored prevention or intervention strategies for adolescents following distinct joint developmental trajectories of Internet addiction and depressive symptoms during their junior middle school years.

## Methods

### Participants and procedure

Quantitative surveys were administered to participants in October and November 2016 (T1; 1st semester of Grade 7), October and November 2017 (T2; 1st semester of Grade 8), and October 2018 (T3; 1st semester of Grade 9) in Shenzhen, Guangdong Province, China. Five districts of Shenzhen, which encompasses nine administrative regions, were randomly selected for the study. Within each selected district, one school was designated as the research focal point, leading to the inclusion of five secondary schools in total. Participants from the five schools were selected based on three criteria: (1) enrollment in the seventh grade, (2) obtaining permission from their legal guardians to participate, and (3) voluntary consent to be involved. In this study, we chose to assess participants collectively in the classroom, ensuring that all students completed the assessment under consistent conditions. Adolescents were guided to complete pen-and-paper questionnaires individually. A total of 1,544 adolescents with a mean age of 12.46 years old (SD = 0.63) participated in the first data collection. A total of 1,511 adolescents participated in the second data collection. A total of 1,480 adolescents participated in the third data collection. After cleaning the invalid data, 1,301 adolescents completed all three assessments. Among them, 666 (51.2%) were boys, 621 (47.7%) were girls, and 14 (1.1%) did not report their gender. See Table 1 for further demographic details regarding the participants. The study was conducted with the approval of both the Human Research Ethics Committee of the corresponding author and the administrative committees of the investigated schools. The dataset used in this study has been utilized in prior research, and has been published in several papers (54, 56, 64). The current study offers a novel analysis by focusing on the joint trajectories of Internet addiction and depressive symptoms, a perspective that differs both methodologically and theoretically from our earlier work.

**Table 1:** Demographic characteristics of participants.

Variables	*N* (%)/M (SD)
Age	12.46 (0.63)
**Gender**	
Male	666 (51.19%)
Female	621 (47.73%)
Missing	14 (1.08%)
**Sibling(s)**	
Only child	499 (38.36%)
Not-only child	799 (61.41%)
Missing	3 (0.23%)
**Family structure**	
Intact	1,222 (93.93%)
Non-intact	68 (5.23%)
Missing	11(0.85%)
**Academic**	
Poor	45 (3.46%)
Fair	278 (21.37%)
Good	450 (34.59%)
Very good	408 (31.36%)
Excellent	102 (7.84%)
Missing	18 (1.38%)

### Instruments

#### Internet addiction

We used the Chinese version of Young’s Internet Addiction Test (65) to assess adolescent Internet addiction. The scale includes 10 items in which adolescents react to these items according to whether they had expressed the listed behaviors in the past year (0 = “no,” 1 = “yes”). The total IA scores ranged from 0 to 10. The overall severity was evaluated by calculating the total score of 10 items. In the present study, the scale demonstrated good reliability, with Cronbach’s alphas of 0.76, 0.75, and 0.83 for T1, T2, and T3, respectively. According to previous research, 4 was considered the cutoff score for screening whether an adolescent was identified as having “Internet addiction” (3).

#### Depressive symptoms

The Chinese version of the Center for Epidemiological Studies Depression Scale [CES-D; (66)] was used to measure adolescent depressive symptoms. Prior studies have shown that the CES-D has good psychometric properties for Chinese adolescents (67). When assessed with this scale, adolescents were instructed to indicate how frequently they experienced depressive symptoms in the past week. All items were measured on a 4-point scale (0 = “rarely or none of the time,” 1 = “sometimes,” 2 = “often,” and 3 = “most or all of the time”). The total CES-D scores ranged from 0 to 60, with higher scores indicating more severe depressive symptoms. In our study, the CES-D exhibited good internal consistency, with the Cronbach’s alphas of 0. 85, 0.85, and 0.88 for T1, T2, and T3, respectively. Based on the suggestion of Kroenke and Spitzer (68), we have adopted a cutoff value of 16 for screening individuals with depressive symptoms.

#### Individual factors

Individual variables included gender and psychological competencies. Adolescents reported their gender (“boy” coded as “1,” “girl” coded as “0”). Psychological competencies were measured by the Chinese version of the Positive Youth Development Scale (CPYDS), which consists of 15 positive youth development constructs (69). Adolescents rated the CPYDS items on a 6-point Likert scale ranging from 1 “strongly disagree” to 6 “strongly agree.” A higher total score reflects greater psychological competence. The CPYDS has demonstrated excellent reliability among Chinese adolescent samples (70, 71). The Cronbach’s alpha in this study was 0.95.

#### Family factors

The family variables included family structure, single-child family or not, and family function. Adolescents reported whether they came from a single-child family or not (“only-child family” coded as “1,” “non-only child family” coded as “0”) and intact family or not (“intact family” coded as “1,” “non-intact family” coded as “0”). Family function was assessed with the nine-item Chinese version of the Family Assessment Instrument [CFAI; (72)]. Items were evaluated on a 5-point scale (1 = “very dissimilar” to 5 = “very similar”). The Cronbach’s alpha for family function in the present study was 0.86. The scores for nine items were averaged, with higher scores representing healthier family function.

#### School factors

The school variable included academic performance. Academic performance was assessed with a single item. Adolescents provided their answers to the question about their relative academic performance within their own class on a 5-point scale (1 = “poor” to 5 = “excellent”) (3). In this study, higher scores indicate greater academic performance.

### Statistical analysis

We calculated the mean, standard deviations and correlations between Internet addiction and depressive symptoms across time points. Then, using the Mplus 8.0 software (73), we applied linear unconditional parallel-process latent class growth modeling (PPLCGM) to distinguish the joint longitudinal trajectories of Internet addiction and depressive symptoms from early adolescence (Grade 7) to middle adolescence (Grade 9). PPLCGM is an extension of the traditional latent class growth analysis (LCGA) that allows for the simultaneous consideration of multiple growth trajectories (74). This approach is particularly suitable for our research questions because it enables us to model the joint developmental processes of Internet addiction and depressive symptoms, as well as to identify distinct subgroups of individuals following similar trajectories over time. In reference to previous similar studies (74–76), the resulting classes of joint trajectories were compared and selected according to a series of fitting indices: (1) the Akaike Information Criterion (AIC), the smallest value represents the most parsimonious model; (2) the Bayesian Information Criterion (BIC), the smallest value presents the most parsimonious model; (3) the adjusted Bayesian Information Criterion (aBIC), the smallest value indicates the most parsimonious model; (4) the Lo–Mendell–Rubin Likelihood Ratio test (LMR-LRT), the model with significant *p*-values of LMR-LRT represents a better fit than the model with one less class; (5) the adjusted LMR-LRT (ALMR-LRT), the model with significant *p*-values of ALMR-LRT reflects a better fit than the model with one less class; (6) the Bootstrapped Likelihood Ratio test (BLRT), the model with significant *p*-values of BLRT represents a better fit than the model with one less class; and (7) the entropy, values ranging from 0 to 1, closer to 1 implies more accurate classification. The selection of the best-fitting solutions was based on theoretical coherence, including the substantive interpretability of the trajectory classes and the identification of trajectories without overfitting, as well as explanatory relevance (75). Additionally, we considered whether each class size comprised at least 5% of the sample (76). After that, we conducted a multinomial logistic regression to assess the role of various individual, family, and school variables in predicting joint trajectory class memberships.

## Results

### Descriptive statistics and correlations of variables

As Table 2 shows, the means and standard deviations for Internet addiction at each measurement wave were 1.55 (1.99), 1.62 (1.99), and 1.47 (2.13) for Time 1 to Time 3, respectively. The means and standard deviations for depressive symptoms at Time 1 to Time 3 were 13.66 (9.15), 13.76 (9.33), and 12.40 (9.31), respectively. The correlations across the three time points ranged from 0.21 to 0.39 for Internet addiction, and from 0.25 to 0.42 for depressive symptoms. At the same measurement wave, the correlations between Internet addiction and depressive symptoms were 0.33 for T1, 0.36 for T2, and 0.44 for T3, respectively. Additionally, the correlations between gender and T1 Internet addiction and T3 depressive symptoms were significant, and single-child family and family structure were both significantly associated with depressive symptoms at T2 and T3. Moreover, the correlations of predictors (positive youth development, family function, and academic performance) at T1 with Internet addiction at T1, T2, and T3 were statistically significant. Likewise, these predictors at T1 were also significantly associated with depressive symptoms at T1, T2, and T3. More details are presented in Table 2.

**Table 2:** Descriptive statistics and correlation analyses among Internet addiction, depressive symptoms, and predictive variables.

Variables	1	2	3	4	5	6	7	8	9	10	11	12
1. T1IA	1											
2. T2IA	0.25^***^	1										
3. T3IA	0.39^***^	0.21^***^	1									
4. T1Dep	0.33^***^	0.16^***^	0.18^***^	1								
5. T2Dep	0.10^***^	0.36^***^	0.11^***^	0.25^***^	1							
6. T3Dep	0.25^***^	0.19^***^	0.44^***^	0.42^***^	0.25^***^	1						
7. Gender	0.08^**^	0.04	−0.05	−0.03	−0.01	−0.11^***^	1					
8. PYD	−0.28^**^	−0.16^***^	−0.15^***^	−0.45^***^	−0.18^***^	−0.29^***^	−0.03	1				
9. Sinbing	−0.05	−0.01	0.01	−0.09^**^	−0.07^*^	−0.01	0.07^*^	0.06^*^	1			
10. FS	−0.02	−0.04	−0.05	−0.06^*^	−0.10^***^	−0.04	0.00	0.09^**^	−0.06^*^	1		
11. FF	−0.25^***^	−0.16^***^	−0.18^***^	−0.37^***^	−0.19^***^	−0.29^***^	−0.04	0.52^***^	0.11^***^	0.18^***^	1	
12. AP	−0.14^***^	−0.09^**^	−0.10^***^	−0.21^***^	−0.11^***^	−0.16^***^	−0.03	0.27^***^	0.13^***^	0.00	0.18^***^	1
*M*	1.55	1.62	1.47	13.66	13.76	12.40	-	4.72	-	-	4.07	3.19
*SD*	1.99	1.99	2.13	9.15	9.33	9.31	-	0.68	-	-	0.77	0.98

T1, Time 1 (Grade 7); T2, Time 2 (Grade 8); T3, Time 3 (Grade 9); IA, Internet Addiction; Dep, Depressive Symptoms; PYD, Positive Youth Development; FS, Family Structure; FF, Family Function; AP, Academic Performance. ^*^p < 0.05; ^**^p < 0.01; ^***^p < 0.001.

### Joint trajectories of Internet addiction and depressive symptoms

We computed 1- to 6-class solutions for joint trajectory groups of Internet addiction and depressive symptoms. The model fit indices for these solutions are summarized in Table 3. The results showed that the values of AIC, BIC, and aBIC decreased from 1- to 6-class solutions, and these classes of solutions all had acceptable entropy (>0.80). We found significant BLRT in 2- to 6-classes, while the LMR-LRT and ALMR-LRT results suggested that the two-, three- and four-class solutions were suitable. Further observation confirmed that the 5-class to 6-class solution should be discarded because they included a subgroup comprising less than 5% of the total participants. Given that the model fit of the optimal trajectory class is indicated by smaller values of AIC, BIC, and aBIC, and a significant result of LMR-LRT, ALMR-LRT, and BLRT, as well as a higher entropy, after balancing the criteria of model fit indices, we considered the 4-class model of joint trajectory groups as the fittest solution for our data. As mentioned above, we chose 4 as the cutoff value for screening for Internet addiction, and 16 for depressive symptoms. After integrating multiple indicators of intercepts, slopes, and cutoff scores for Internet addiction and depressive symptoms, we labeled the four joint trajectory classes as the Health Group, Comorbidity-Worsening Group, Asymptomatic-Comorbid Risk Group, and Prominent Depressive Symptoms-Remission Group.

**Table 3:** Model fit indices for 1- to 6- class solutions for Internet addiction and depressive symptoms.

Class	AIC	BIC	aBIC	Entropy	LMR-LRT	ALMR-LRT	BLRT	membership percentages (%)
1-Class	45113.84	45165.55	45133.78					
2-Class	44084.93	44162.49	44114.84	0.91	0.0004	1010.71 (0.0005)	<0.001	0.872/0.128
3-Class	43810.96	43914.38	43850.85	0.84	0.0016	276.263 (0.0018)	<0.001	0.772/0.143/0.085
**4**-Class	**43621.31**	**43750.58**	**43671.17**	**0.85**	**0.0088**	**194.235 (0.0099)**	**<0.001**	**0.701/ 0.065/ 0.114/0.120**
5-Class	43452.13	43607.26	43511.96	0.87	0.1221	174.317 (0.1291)	<0.001	0.072/0.703/0.034/0.107/0.084
6-Class	43312.25	43493.23	43382.05	0.88	0.2405	145.816 (0.2496)	<0.001	0.123/0.056/0.099/0.663/0.040/0.019

AIC, Akaike Information Criterion; BIC, Bayesian Information Criterion; aBIC, adjusted Bayesian Information Criterion; LMR-LRT, Lo–Mendell–Rubin Likelihood Ratio test; ALMRLRT, adjusted Lo–Mendell–Rubin Likelihood Ratio test; BLRT, Bootstrapped Likelihood Ratio test. Bold indicates best fit.

**Figure 1:**
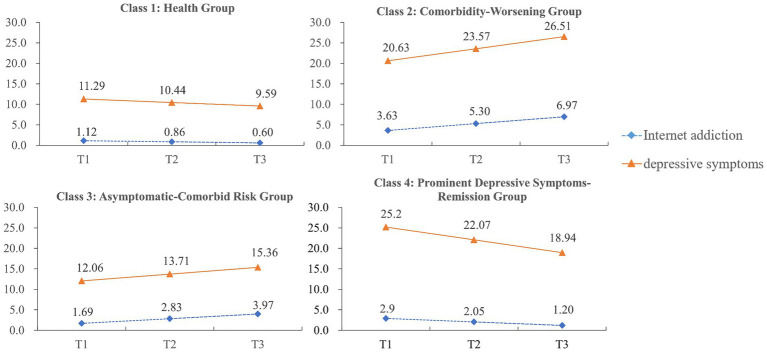
Estimated means of Internet addiction and depressive Symptoms in PLCGM. Y-axis indicates the means of Internet addiction/depressive symptoms; X-axis indicates the measurement times. T1–T3 represents Time 1–Time 3, respectively.

The first trajectory class (Health Group) included 70.1% of the adolescents (*n* = 912) with no or very few Internet addiction symptoms as well as depression episodes in the first assessment (intercepts =1.12 and 11.29, respectively; *p* < 0.001). Moreover, both Internet addiction and depressive symptoms slightly decreased throughout the three assessments (slopes = −0.26 and − 0.85, respectively; *p* < 0.001), with both scores being consistently below the cutoff value during the study period. The second trajectory class (Comorbidity-Worsening Group) comprised 6.5% of the adolescents (*n* = 85) with high levels of Internet addiction and depressive symptoms at baseline, and both increased rapidly and significantly during the three-year period. The Internet addiction score in this class was initially nearly the cutoff (intercept = 3.63, *p* < 0.001), and increased at a mean linear rate of 1.67 points at each measurement time (slope = 1.67, *p* < 0.001). The depressive symptoms in this class were highly similar to Internet addiction, with scores above the cutoff value at baseline (intercept = 20.63, *p* < 0.001) and a significantly positive mean slope value (slope = 2.94, *p* = 0.006). The third trajectory class (Asymptomatic-Comorbid Risk Group) included 11.4% of the adolescents (n = 148) with low initial scores (below the cutoff value) for both Internet addiction and depressive symptoms (intercepts = 1.69 and 12.06, respectively; *p* < 0.001). However, in this latent class, the Internet addiction and depressive symptom scores continually increased at the two subsequent assessment points (slopes = 1.14 and 1.65, respectively; *p* < 0.001), and depressive symptom scores ultimately increased nearly to the diagnostic criteria at the last assessment point. The fourth trajectory class (Prominent Depressive Symptoms-Remission Group) consisted of 12.0% of the adolescents (n = 156) with high Internet addiction scores (intercepts = 2.90, *p* < 0.001) and the highest depressive symptom scores (intercepts = 25.20, *p* < 0.001) at Time 1, both of which decreased during the three junior years. Depressive symptom scores in this class displayed a very rapid decline (slope = −3.13, *p* < 0.001), but scores were persistently higher than the cutoff score of 16 at each assessment point. Meanwhile, Internet addiction scores in this group decreased quite slowly (slope = −0.85, *p* < 0.001), and the scores were consistently lower than the cutoff score of 4 across the three assessments. Figure 1 shows the estimated growth parameters and plots for each of the four classes, respectively.

### Predictor of the joint trajectory groups of Internet addiction and depressive symptoms

In consideration of the clinical intervention, in the current study, three groups with symptoms (Comorbidity-Worsening Group, Asymptomatic-Comorbid Risk Group, and Prominent Depressive Symptoms-Remission Group) should be the focus in comparing with the Health Group. Thereby, to offer effective and targeted interventions for these groups, we probed the role of individual factors (gender, positive youth development), family factors (family structure, whether come from only-child family, family function) and school factors (academic performance) in predicting the membership in the Comorbidity-Worsening Group, Asymptomatic-Comorbid Risk Group, and Prominent Depressive Symptoms-Remission Group, by comparing each of them with the Health Group. The results of the multinomial logistic regression analysis of these individual, family and school predictors are displayed in Table 4.

**Table 4:** Predictive effect of membership in the joint trajectory groups with the health group as the reference category.

Predictors	Reference = *Health Group*		
	*Comorbidity-Worsening Group* vs. *Health Group*	*Asymptomatic-Comorbid Risk Group* vs. *Health Group*	*Prominent Depressive Symptoms-Remission Group* vs. *Health Group*
**Individual variable**			
Gender	0.59^*^[0.34, 1.03]	0.63^**^[0.41, 0.98]	0.91 [0.55, 1.49]
PYD	0.39^***^[0.24, 0.62]	0.57^***^[0.40, 0.81]	0.32^***^[0.21, 0.50]
**Family variable**			
Sibling	1.75 [1.00, 3.05]	0.98 [0.62, 1.56]	0.65 [0.37, 1.12]
FS	1.36 [0.37, 5.042]	0.60 [0.25, 1.43]	0.64 [0.24, 1.68]
FF	0.42^***^[0.30, 0.59]	0.75^*^[0.54, 1.03]	0.42^***^[0.30, 0.58]
**School variable**			
Aca	0.64^***^[0.47, 0.85]	1.10 [0.87, 1.40]	0.81 [0.61, 1.07]

*N* = 1,301. OR, odds ratio. ^*^p < 0.05; ^**^p < 0.01; ^***^p < 0.001. Gender (male = 1, female = 0); Family Structure (Intact = 1, Non-intact = 0); Sibling (Only child = 1, Not-only child = 0). PYD, Positive Youth Development; FS, Family Structure; FF, Family Function; AP, Academic Performance.

Using the Health Group as the reference category, our results showed that family structure and whether come from only-child family had nonsignificant associations with distinct co-developmental trajectories of adolescent Internet addiction and depressive symptoms. However, gender, positive youth development, family function, and academic performance were significantly related to the three joint trajectories groups. To be specifically, compared to those in the Health Group, the participants in the Comorbidity-Worsening Group and Asymptomatic-Comorbid Risk Group were less likely to be boys (OR = 0.59 and 0.63, respectively). Moreover, compared with those in the Health Group, having more positive youth development and healthier family function both significantly decreased the odds of following the Comorbidity-Worsening Group (OR = 0.39 and 0.42, respectively), Asymptomatic-Comorbid Risk Group (OR = 0.57 and 0.75, respectively), and Prominent Depressive Symptoms-Remission Group (OR = 0.32 and 0.42, respectively). Additionally, reporting better academic performance significantly decreased the odds of following the Comorbidity-Worsening Group (OR = 0.64) only.

## Discussion

Adopting a three-year longitudinal design combined with parallel-process latent class growth modeling, the present study addressed the co-developmental patterns of Internet addiction and depressive symptoms from early adolescence to middle adolescence, as well as the potential predictors related to individual, family and school variables. The findings revealed four distinct co-developmental patterns: Health Group, Comorbidity-Worsening Group, Asymptomatic-Comorbid Risk Group, and Prominent Depressive Symptoms-Remission Group. Furthermore, the predicted effects of gender, positive youth development, family function, and academic performance on the membership in the established joint trajectory subgroups were also endorsed by this study.

### Joint developmental trajectories of Internet addiction and depressive symptoms among adolescents

As our findings revealed, Internet addiction and depressive symptoms jointly changed in a heterogeneous way from early adolescence to middle adolescence. Our findings indicated four different co-developmental trajectories of the two types of mental health concerns: Health Group, Comorbidity-Worsening Group, Asymptomatic-Comorbid Risk Group, and Prominent Depressive Symptoms-Remission Group. Within each subgroup, adolescents generally exhibited similar developmental patterns across two mental health issues, which supporting the idea that Internet addiction and depressive symptoms are inextricably linked (9, 77). However, the initial level of Internet addiction and depressive symptoms (i.e., low-low/high-high), growth patterns of Internet addiction and depressive symptoms (i.e., increasing/decreasing), and predominant symptoms (i.e., comorbidity, asymptomatic, and depressive symptoms only) varied across adolescents in different subgroups, providing additional support for heterogeneous joint trajectories of Internet addiction and depressive symptoms.

The present study successfully identified three congruent subgroups that exhibited comparable initial levels and consistent developmental patterns in terms of both Internet addiction and depressive symptoms (i.e., Health Group, Comorbidity-Worsening Group and Asymptomatic-Comorbid Risk Group). First, consistent with prior studies showing that most adolescents exhibit low or no Internet addictive behaviors (78) and depressive symptoms (79), we found that the majority of adolescents (70.1%) exhibited low levels of Internet addiction and depressive symptoms and remained healthy (the Health Group) throughout their three-year tenure in junior middle school. Our findings were also in line with previous developmental studies of Internet addiction (21, 22) and depressive symptoms (26).

Second, our research identified a Comorbidity-Worsening Group, comprising 6.5% of the sample population. Within this group, both Internet addiction and depressive symptoms started at relatively high levels and then increased over time. The developmental pattern of this subgroup is in accordance with the findings of studies showing that Internet addiction and depressive symptoms could exhibit a vicious cycle. The reason may be that the presence of Internet addiction among adolescents may contribute to the manifestation of depressive symptoms, subsequently reinforcing their inclination toward increased Internet engagement (18, 80, 81). Such reciprocal interaction perpetuates a self-reinforcing cycle, thereby engendering deleterious progression. Therefore, adolescents with co-morbid Internet addiction and depressive symptoms appear to be at a vulnerable stage and are experiencing a deterioration in development due to the mutually malignant effects of Internet addiction and depressive symptoms. This, to some extent, supports both the compensatory Internet use theory (15) and the social displacement hypothesis (16), which is also consistent with recent research implying the existence of shared neurobiological mechanisms, such as oxytocinergic and dopaminergic expression, between Internet addiction and depressive symptoms (82, 83). Notably, this finding emphasizes that the initial year of junior high school is a particularly critical period for detecting Internet addiction and depressive symptoms, as well as a pivotal opportunity for preventing the deterioration of Internet addiction and depressive symptoms. Additionally, these findings may provide valuable insights to scholars and clinicians that the earlier the intervention for Internet addiction and depressive symptoms is, the more effective it might be at mitigating them among adolescents.

Third, our study identified an Asymptomatic-Comorbid Risk Group (11.4%), in which Internet addiction and depressive symptoms were initially low but both increased rapidly across time. For this subgroup of adolescents, an increasing level of depressive symptoms reflects the inadequacy of emotion regulation strategies (84, 85); addictive behaviors into the Internet could serve as a means to help adolescents escape unpleasant emotions. It seems that the transition from childhood to adolescence represents a vulnerable period for the onset and escalation of both depressive symptoms and Internet addiction, as individuals may progress from being initially asymptomatic to having a co-occurring risk. The challenging process necessitates behavioral adjustments to cope with various biological, psychological, and social changes (86, 87). These adjustments may entail exposure to stressors and potentially traumatic experiences, which may trigger depressive symptoms and Internet addiction. Overall, the identification of the three congruent groups (i.e., Health Group, Comorbidity-Worsening Group and Asymptomatic-Comorbid Risk Group) suggested that Internet addiction and depressive symptoms exhibited similarities in developmental status and temporal trends among the majority of middle school students. Consequently, integrated interventions targeting both Internet addiction and depressive symptoms are recommended.

Regarding the Prominent Depressive Symptoms-Remission Group (12.0%), our results showed that depressive symptoms in this subgroup were at the highest level and then showed a rapid decreasing trend with time, whereas Internet addiction was at a relatively moderate level and exhibited a gradual decline over time, but did not reach the threshold of addiction disorder during the 3 years. Nonetheless, in this study, we did not find a subgroup characterized by elevated levels of Internet addiction alongside low levels of low depressive symptoms. Our findings support the idea that Internet addiction commonly occurs concomitantly with depressive symptoms, while depressive symptoms tend to manifest independently. This finding aligns with the outcomes reported in several prior research, which found that Internet addiction may result in depressive symptoms, rather than vice versa (88, 89). By intensive tracking over short periods of time, future researchers should further examine whether higher levels of Internet addiction indeed co-occur with depressive symptoms, or whether higher levels of Internet addiction may occur without depressive symptoms.

### Predictor of the joint trajectories of Internet addiction and depressive symptoms

In the present study, after controlling for the effects of multiple factors, we found that positive youth development and family function significantly reduced the risk of membership in the Comorbidity-Worsening Group, Asymptomatic-Comorbid Risk Group and Prominent Depressive Symptoms-Remission Group compared to the Healthy Group. Our findings were in accordance with previous related studies on the relationship between positive youth development, family function and two forms of mental health problems (i.e., Internet addiction and depressive symptoms) (22, 90). This can be explained by development assets theory (91), which assumes that the developmental resources (both external and internal) that adolescents possess will facilitate effective coping with environmental stressors, thus minimizing the occurrence of various mental health problems (71, 91, 92). Related studies have further confirmed that adolescents are more likely to develop well and have fewer addiction and depression problems when they have access to a variety of resources. Thus, those who exhibit high levels of positive youth development are less likely to be plagued by Internet addiction and depressive symptoms.

In the present study, positive youth development was identified as an internal asset, that is an important predictor of adolescent mental health and a crucial protective factor against adolescent problem behaviors (47, 93). During adolescence, high levels of positive youth development are effective at fostering adolescents’ internal self-regulation (94) and could encourage individuals to effectively address encountered challenges, thereby substantially diminishing the probability of exacerbating Internet addiction (35, 39, 95) and depressive symptoms (96, 97). Family function, as an important external resource for adolescent development, has a significant impact on preventing the onset and continuity of adolescents’ mental health problems (98). This finding aligns with a substantial body of prior research investigating the impact of family on adolescents. These studies have documented that healthy family functioning, characterized by strong family cohesion, a warm family environment, and constructive parent–child interactions, is conducive to the healthy development of adolescents and substantially diminishes the likelihood of adolescent Internet addiction (99–102) and depressive symptoms (56, 103). Notably, in this study, positive youth development and family functioning were significantly associated with all three groups (the Comorbidity-Worsening Group, Asymptomatic-Comorbid Risk Group, and Prominent Depressive Symptoms-Remission Group) compared with the Healthy Group, confirming the predictive effect of positive youth development and family function on both the initial level and developmental trends of Internet addiction and depressive symptoms. To some extent, our study extends previous studies by indicating that positive youth development and family function not only have transient but also long-term effects on adolescents’ Internet addiction and depressive symptoms.

In addition, we found that low academic performance in early adolescence increased the likelihood of belonging to the Comorbidity-Worsening Group compared to adolescents in the Health Group. This finding is consistent with the outcomes of a recent investigation conducted on middle school students in China, which revealed that high academic performance was indicative of low-risk depressive symptom trajectories (104). Furthermore, it partially supports the academic incompetence hypothesis (59), in which failure in academic performance erodes individuals’ intrinsic resources, decreasing their ability to cope with addiction and depression. Thus, challenged by academic failure, these co-morbid adolescents may experience more severe distress, which may trigger a worsening of Internet addiction and depressive symptoms. It is worth noting that the present study found that the effects of poor academic performance on the Asymptomatic-Comorbid Risk Group and the Prominent Depressive Symptoms-Remission Group were not significant. This finding implies that the effects of poor academic performance are significant solely when Internet addiction and depressive symptoms are both at high risk in the initial state of adolescence. For adolescents with relatively low levels of depressive symptoms and Internet addiction (the Asymptomatic-Comorbid Risk Group), and for adolescents with only high levels of depressive symptoms but no Internet addiction (the Prominent Depressive Symptoms-Remission Group), the impact of academic underachievement is not proximal enough to compromise functioning.

Another noteworthy finding was that girls were significantly more likely to belong to the Comorbidity-Worsening Group and the Asymptomatic-Comorbid Risk Group than were boys. This outcome aligns with prior research showing that the adverse consequences of Internet use on depression are more severe for females than for males (105), while the prevalence of Internet addiction is greater among males than females (106). One reasonable explanation is that this discrepancy may be attributed to divergent biological changes and developmental rates between boys and girls (107). Specifically, during puberty, surges in estrogen and other hormones in girls can potentially disrupt internal homeostasis. Consequently, girls may exhibit heightened sensitivity to psychosocial factors such as stress (107). This heightened vulnerability may contribute to overindulgence in the Internet and exacerbate internalized issues (e.g., depression), surpassing those experienced by boys (108, 109).

To summarize, the present study has identified positive youth development, family function, academic performance, and gender as significant predictors of the joint developmental trajectories of Internet addiction and depressive symptoms among adolescents. Our research has determined that certain variables exert a similar and universally applicable influence. Specifically, our findings underscore the robust influence of positive youth development and family functioning both as protective factors in distinguishing between the Comorbidity-Worsening Group, Asymptomatic-Comorbid Risk Group, Prominent Depressive Symptoms-Remission Group, and the Health Group, in accordance with the development assets theory. Positive youth development fosters internal self-regulation, and healthy family function provides external support, both of which contribute to the trajectory with both immediate and enduring implications for adolescent mental health. Additionally, our study identified particular variables, noting that poor academic achievement could only differentiate the Comorbidity-Worsening Group from the Health Group, while gender enables the differentiation of the Comorbidity-Worsening Group, Asymptomatic-Comorbid Risk Group, and the Health Group. Understanding these complex relationships could inform the development of more nuanced and effective preventive measures.

### Limitations, strengths, and implications

Several limitations should be noted. First, although this study investigated Internet addiction and depressive symptoms from early to middle adolescence, it failed to track the progression of these symptoms throughout adolescence. Consequently, further studies are needed to validate and broaden these findings by encompassing a wider age range (i.e., primary, middle, to high school) to gain a comprehensive understanding of the long-term co-trajectories of Internet addiction and depressive symptoms. Second, our study exclusively examined the self-reported measures of Internet addiction and depressive symptoms. Therefore, it is important to acknowledge that participants’ responses might have been influenced by social desirability bias and self-presentation concerns, which could introduce common method bias. Future researchers are encouraged to gather data from multiple sources such as peers, teachers, and parents. Third, the current study has not fully accounted for stable individual characteristics that may influence the development of both Internet addiction and depressive symptoms. For instance, we did not consider the potential role of boredom proneness, which, as suggested by recent literature, could be a significant predictor of both depressive symptoms and Internet addiction (110, 111). Future research should integrate measures of boredom proneness and other stable individual differences to enhance the understanding of these complex dynamics. Fourth, the annual intervals between assessments may have allowed for the influence of significant life events, potentially introducing variability in the reported Internet addiction and depressive symptoms. These life events, such as changes in family structure, academic pressure, or social relationships, might have affected the mental health trajectories of our participants in ways that our study design does not fully capture. Further research with more frequent assessment points, including an evaluation of life events, is necessary to better understand these dynamics. Finally, our data were collected between 2016 and 2018. Given the rapid pace of technological change and the evolving nature of adolescent behaviors and mental health, some aspects of our findings might not fully reflect the current state of the variables under investigation. Nevertheless, we believe that the longitudinal design of our study and the robust co-development of Internet addiction and depressive symptoms might provide valuable insights. Future studies may benefit from incorporating more recent data to assess the ongoing development of these mental health concerns and to validate the generalizability of our findings in the current context.

Despite the aforementioned limitations, several important strengths emerged from the present study. First, this study utilized a longitudinal design of three waves covering the transition from early adolescence through middle adolescence, providing new insight into the joint developmental process of Internet addiction and depressive symptoms in this crucial developmental period. Second, by employing a person-centered, multi-trajectory approach, our study detected heterogeneous co-developmental trajectories of two prevalent psychological issues (Internet addiction and depressive symptoms) within a substantial cohort of Chinese adolescents, contributing to a profound and comprehensive understanding of the intricate nature of the concurrent developmental patterns of Internet addiction and depressive symptoms. Third, this study examined how individual, family, and school factors might relate to the co-developmental trajectories of Internet addiction and depressive symptoms. Such analyses advanced the existing knowledge on the risk and protective factors associated with these co-developmental patterns, and thereby enhanced our understanding of how adolescents’ Internet addiction and depressive symptoms could be effectively prevented.

The findings of our study hold significant implications for the evaluation, prevention, and intervention of Internet addiction and depressive symptoms among adolescents. The identification of four distinct subgroups implies that prevention and intervention approaches for adolescent Internet addiction and depressive symptoms should adopt different focal points and targeted strategies tailored to the specific co-developmental patterns of adolescents. On the one hand, the discovery of the Comorbidity-Worsening Group and Asymptomatic-Comorbid Risk Group revealed the persistent co-occurrence of Internet addiction and depressive symptoms in adolescents, suggesting that interventions for Internet addiction and depressive symptoms should be integrated. On the other hand, the detection of distinct co-development trajectories of Internet addiction and depressive symptoms implies that customized interventions targeting these two mental health issues may yield optimal outcomes for diverse individuals. For example, interventions that primarily address alleviating depressive symptoms would be most suitable for the Prominent Depressive Symptoms-Remission Group.

Furthermore, early intervention efforts should be tailored based on group membership, given that protective and risk factors and their combinations vary across concomitant trajectories of Internet addiction and depressive symptoms. For example, after taking into account the effects of individual, family, and school factors, positive youth development and family functioning emerged as significant protective factors linked to the Comorbidity-Worsening Group, Asymptomatic-Comorbid Risk Group and Prominent Depressive Symptoms-Remission Group. Thus, it is imperative for schools and families to collaborate effectively to cultivate junior high school students’ intrinsic positive resources, such as competence, connection, resilience, and confidence, to better address the issue of Internet addiction and depressive symptoms among adolescents. Meanwhile, preventive initiatives should focus on promoting family cohesion, cultivating a harmonious family atmosphere, and reducing family discord to make the family function well. In addition, poor academic performance was a significant risk factor associated with the Comorbidity-Worsening Group. This finding suggests that parents and teachers should keep close tabs on adolescents with co-morbid Internet addiction and depressive symptoms. Particularly when adolescents underperform academically, teaching emotion regulation strategies and monitoring online activities should be provided in a timely manner to reduce the co-worsening of Internet addiction and depressive symptoms in adolescents.

## Data availability statement

The raw data supporting the conclusions of this article will be made available by the authors, without undue reservation.

## Ethics statement

The studies involving humans were approved by the Human Research Ethics Committee of Shenzhen University. The studies were conducted in accordance with the local legislation and institutional requirements. Written informed consent for participation in this study was provided by the participants’ legal guardians/next of kin.

## Author contributions

JZ: Conceptualization, Data curation, Formal analysis, Investigation, Writing – original draft. EW: Conceptualization, Data curation, Formal analysis, Investigation, Writing – original draft. LZ: Funding acquisition, Project administration, Writing – review & editing. XC: Funding acquisition, Project administration, Writing – review & editing.
